# Analysis of medical malpractice litigation following vertebral augmentation therapy in China from 2008 to 2018

**DOI:** 10.1097/MD.0000000000030972

**Published:** 2022-10-14

**Authors:** Dong Hu, Huawei Liu, Bei Yuan, Suxi Gu, Kai Xu, Fei Song, Songhua Xiao

**Affiliations:** a Department of Orthopaedics, Beijing Tsinghua Changgung Hospital, School of Clinical Medicine, Tsinghua University, Beijing, P.R. China; b Institute for Precision Medicine, Tsinghua University, Beijing, P.R. China.

**Keywords:** China, litigation, malpractice, PKP, PVP, vertebral augmentation therapy

## Abstract

The first case of vertebral augmentation therapy in mainland China was reported in 2000. Since then, it has been widely used in China as a minimally invasive procedure to treat vertebral compression fractures. However, the characteristics of malpractice litigation involving vertebral augmentation therapy remains unclear. This study aims to analyze the characteristics of medical malpractice litigation involving vertebral augmentation therapy in mainland China for the past 10 years. Two online legal databases were queried for court verdicts involving vertebral augmentation therapy from Jan 2009 to Dec 2018 in mainland China. Each case file was then thoroughly reviewed and data pertaining to defendants, plaintiffs, case outcomes, allegations, and verdicts were abstracted, and descriptive analyses were performed. Level of evidence: LEVEL III. A total of 96 cases were enrolled for final analysis. The number of claims increased by five times during the past 10 years. More than two thirds (67.7%, n = 65) of the cases underwent percutaneous vertebroplasty, and 22.9% (n = 22) underwent percutaneous kyphoplasty, the rest (9.4%, n = 9) remained undefined. Paralysis was alleged in 35.4% of cases (n = 34), followed by significant physical injury (34.4%, n = 33). Cement leakage to spinal canal (44.8%, n = 43) is the most commonly cited reason for litigation, followed by incomplete informed consent (42.7%, n = 41), accidental dural puncture (20.8%, n = 20), unsatisfactory clinical outcome (18.8%, n = 18), and misdiagnosis (12.5%, n = 12). Acute pulmonary cement embolism (4.2%, n = 4), wrong-level vertebrae procedure (3.1%, n = 3) and postoperative infection (2.1%, n = 2) were less common causes for concern. Doctors successfully defended themselves only in 8 (8.3%) cases, which resulted in no indemnity payment. The rest 88 (91.7%) cases were closed with a mean verdict payout of 361,580 Yuan (51,654 US dollars). There is a quickly rising trend in the number of medical malpractice litigation involving vertebral augmentation therapy in China. Identifying the most common reasons for litigation and summarizing their characteristics may help decrease litigation rate and improve the patient experience.

## 1. Introduction

Medical malpractice lawsuits pose economic and emotional burdens that weigh heavily on both the physician and the patient. Recent trends showing incidence of medical malpractice litigation in China is increasing rapidly.^[1]^

Vertebral augmentation therapy emerged in the 1990s as a surgical approach to reduce pain and deformity after a vertebral fracture.^[2]^ As currently practiced, vertebral augmentation therapy includes either percutaneous vertebroplasty (PVP), where bone cement is injected percutaneously into the vertebral body, or balloon kyphoplasty (PKP), where a balloon or bone tamp is introduced into the vertebral body, inflated, and then injected with bone cement. The first reported case of vertebral augmentation therapy in mainland China was published in 2000.^[3]^ Despite of the controversial benefits of vertebral augmentation therapy, this technique became popular among mainland China during the past 2 decades.^[4]^

Prior studies have explored medical malpractice litigation related to spine surgery in different countries.^[5–7]^ However, no previous study investigated the malpractice litigation specifically focused on vertebral augmentation therapy. Identifying the most common reasons for litigation and summarizing their characteristics may help decrease litigation rate and improve the patient experience. This study aimed to examine trends and patterns of vertebral augmentation therapy related litigation in mainland China during the past 10 years, using the Wusong and Weike legal databases.

## 2. Methods

Publicly available court verdicts related to vertebral augmentation therapy malpractice litigation in mainland China, from January 2009 to December 2018, were identified in two online legal databases (Westlaw and Weike). These databases provide online legal research service for lawyers and legal professionals which can provide information regarding national cases in China. Court verdicts were obtained using the search terms “vertebroplasty”, “kyphoplasty”, “PVP”, “PKP” and “malpractice”. Exclusion criteria included duplicate cases, unrelated topics, and lack of reported data. Each case file was then thoroughly reviewed and data pertaining to defendants, plaintiffs, case outcomes, allegations, and verdicts were abstracted, and descriptive analyses were performed. Severity of damage was graded from minor/temporary to death. Data analysis was carried out using Excel and Graphpad, and statistical significance was calculated using a two-tailed Student’s *t* test. Ethical approval for this study was waived by Ethics Ccmmittee of Tsinghua Changgung Hospital because this study was conducted on public available data.

## 3. Results

**Figure 1.**
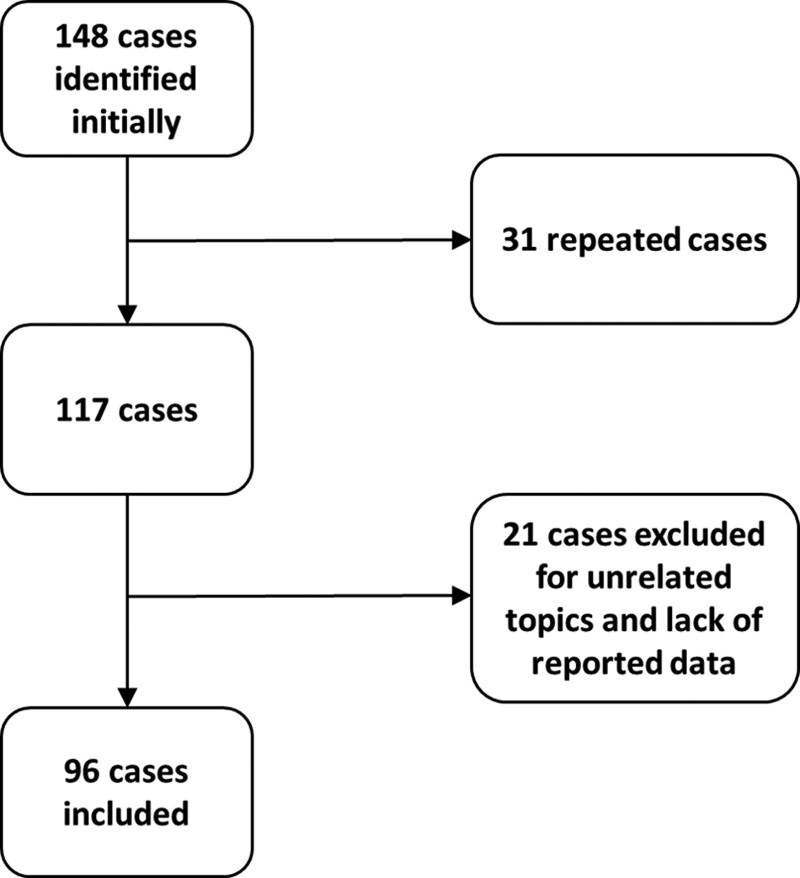
Flowchart describing cases that were included and those that were excluded.

**Figure 2.**
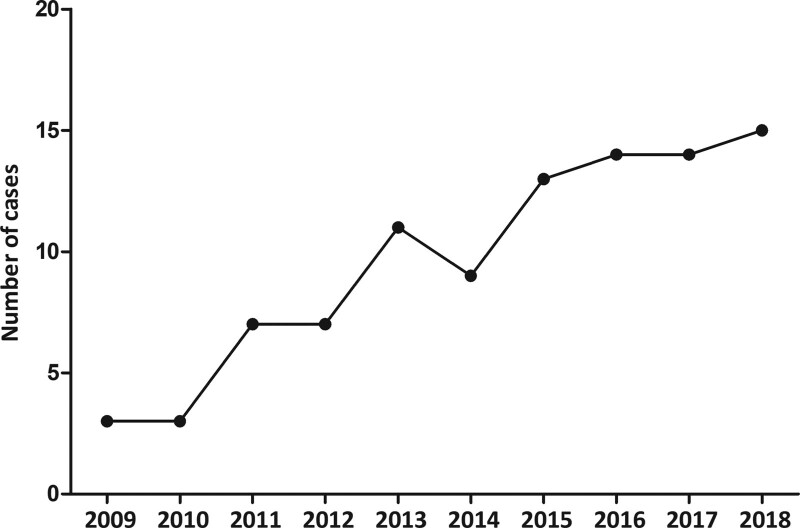
Temporality of claims.

In total, 148 cases were identified from the two databases initially; Thirty-one cases were repeated and 21 cases were excluded for missing information or irrelevance, leaving a total of 96 cases for final analysis (Fig. 1). The number of claims increased by five times during the past 10 years. The year in which claims were judged is displayed in Figure 2.

The mean age of the plaintiffs was 68.6 ± 10.3 years. Thirty cases (31.2%) of the plaintiffs were male and 66 cases (68.8%) were female. Among all the included medical malpractice cases, 72 cases (75.0%) occurred in public hospitals while 24 cases (25.0%) occurred in private ones. It should be noted that the vast majority of medical dispute cases happened in public hospitals were concentrated in tertiary hospitals (70.8%, n = 51).

As shown in Table 1, the most common primary disease leading to treatment is osteoporotic fracture (83.3%, n = 80), followed by trauma (9.4%, n = 9), spinal tuberculosis (5.2%, n = 5), and spinal metastasis tumor (2.1%, n = 2). These pathologic vertebras located most commonly in the thoracolumbar vertebrae (87.5%, n = 84), followed by midthoracic vertebrae (8.3%, n = 8) and lower lumbar vertebrae (4.2%, n = 4). Sixty-eight cases (70.8%) involved single vertebrae during their augmentation therapy, 24 cases (25.0%) involved two vertebras, and 4 cases (4.2%) involved three or even more vertebras. More than two thirds (67.7%, n = 65) of the cases underwent PVP, 22.9% (n = 22) underwent PKP, and the rest (9.4%, n = 9) remained undefined.

**Table 1:** Case characteristics and medical details.

Variable	Value
Sex (male %)	30 (31.2%)
Age	68.6 ± 10.3
Hospital involved (%)	
Public	72 (75.0%)
Private	24 (25.0%)
Primary diseases	
Osteoporotic fracture	80 (83.3%)
Trauma	9 (9.4%)
Spinal tuberculosis	5 (5.2%)
Spinal metastasis tumor	2 (2.1%)
Procedure location	
Midthoracic vertebrae (T5–T8)	8 (8.3%)
Thoracolumbar vertebrae (T11–L2)	84 (87.5%)
Lower lumbar vertebrae (L3–L5)	4 (4.2%)
Number of vertebras involved	
1	68 (70.8%)
2	24 (25.0%)
>2	4 (4.2%)
Operation type	
PVP	65 (67.7%)
PKP	22 (22.9%)
Unidentified	9 (9.4%)
Damage severity (%)	
Minor, Temporary	24 (25.0%)
Significant Physical	33 (34.4%)
Paralysis	34 (35.4%)
Death	5 (5.2%)

PKP = percutaneous kyphoplasty, PVP = percutaneous vertebroplasty.

We further analyzed the disability determination report of patients. Paralysis was alleged in the in the vast majority of cases (35.4%, n = 34), followed by significant physical injury (34.4%, n = 33). Minor or temporary alleged injuries were less common (25.0%, n = 24). Patient death was noted in 5 cases (5.2%).

**Figure 3.**
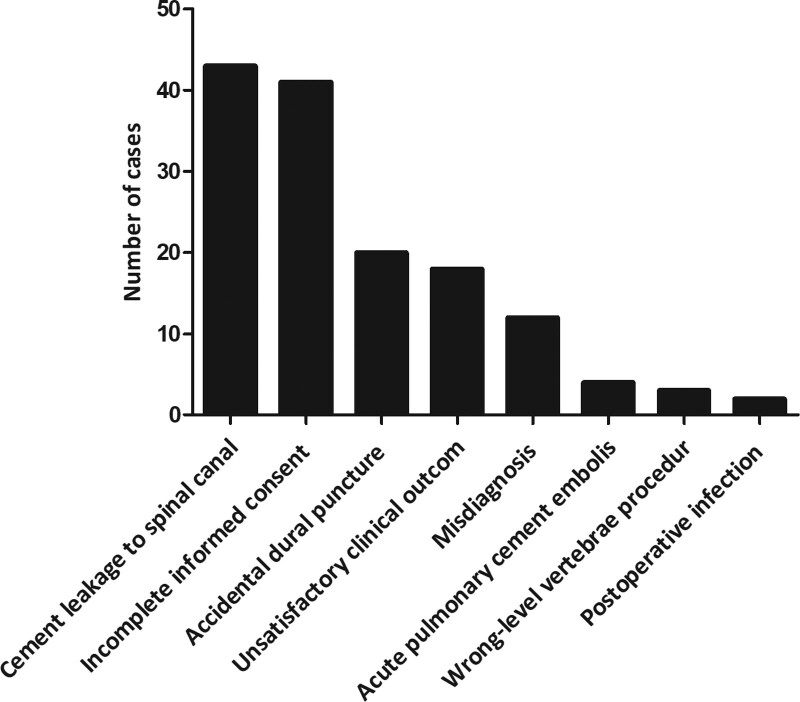
Number of cases for each reason that led to a malpractice claim following vertebral augmentation therapy.

To glean a better understanding of the motivating force behind each claim, an attempt was made to isolate the main issues from the patient’s perspective. As shown in Figure 3, cement leakage to spinal canal (44.8%, n = 43) is the most commonly cited reason for litigation, followed by incomplete informed consent (42.7%, n = 41), accidental dural puncture (20.8%, n = 20), unsatisfactory clinical outcome (18.8%, n = 18), and misdiagnosis (12.5%, n = 12). Acute pulmonary cement embolism (4.2%, n = 4), wrong-level vertebrae procedure (3.1%, n = 3) and postoperative infection (2.1%, n = 2) were less common causes for concern.

A total of 42 cases (43.8%) received additional surgery, among which 36 cases underwent surgical exploration and decompression, 4 cases underwent posterior long segment internal fixation, and 2 cases underwent debridement. Among the 12 cases with the claim of misdiagnosis, there were 6 cases of missing fresh fractured vertebrae, 4 cases of delayed diagnosis of spinal tuberculosis, and 2 cases of delayed diagnosis of spinal metastasis tumor.

The most likely outcome of included litigation was a jury verdict in favor of the plaintiff, as shown in Table 2. Doctors successfully defended themselves only in 8 (8.3%) cases, which resulted in no indemnity payment. The rest 88 (91.7%) cases were closed with a mean verdict payout of 361,580 Yuan (range 15,614–3564,120 Yuan), which was significantly lower than the mean compensation claimed by the plaintiff (921819 Yuan, range 91,023–7248,070 Yuan) (*P* < .05). On average, it took 26.19 months (range 6–90 months) after the date of first operation for the case to end via verdict. Among the cases that surgeons were held not guilty, the time elapsed from the first surgery to the end of verdict was 43 months (range 15–83 months), which is significant lengthier than 24.66 months (range 6–90 months) of guilty (*P* < .05).

**Table 2:** Litigation details.

Verdicts	Mean plaintiff claimed payouts, Yuan (range)	Mean defendant verdict payouts, Yuan (range)	Time from surgery to verdict, mo (range)
Not guilty 8 (8.3%)	202,604 (40,000–574,214)	0 (0–0)	43.00 (15–83)
Guilty 88 (91.7%)	921,819 (91023–7248,070)	361,580 (15,614–3564,120)	24.66 (6–90)
Total 96 (100%)	861,884 (40,000–7248,070)	331,448 (0–3564,120)	26.19 (6–90)

## 4. Discussion

Medical malpractice is a huge problem around the world. The present study was the first one to analyze vertebral augmentation therapy related malpractice claims. A significant year-based increase in the incidence rate of malpractice litigation was observed in current study, which is consistent with the increasing cases of medical malpractice litigation among previous studies from China.^[1,8]^

The present study observed that three-quarter of the enrolled medical litigation cases occurred in public hospitals rather than in private ones. This may be explained by the fact that healthcare services provided by the private sector were far less compared with that from public hospitals in China before 2009, when national reform of health care promoted universal health coverage. We also observed that the vast majority of medical dispute cases were concentrated in tertiary hospitals rather than in lower-level medical settings. A similar striking finding was found in a study by He et al, where tertiary hospitals constituted as high as 59.5% of all medical malpractice cases.^[9]^ The current medical system in China might explain the remarkable number of medical disputes in upper-level healthcare facilities, as China has not yet established a mature dividing system for patients. Patients more likely influx into upper-level healthcare facilities without necessary relation to their disease severity.

Another interesting finding was that more than two thirds of cases in current study involved PVP, much higher than the proportion of PKP involved litigation cases (*P* < .05). This phenomenon may be explained by multiple reasons. First, lots of studies demonstrated that PKP is superior to PVP for its lower cement leakage rate.^[10–12]^ Second, the time when PKP technique entering the China market was later than that of PVP. Third, PKP is less pervasive compared with PVP in China because of its higher material cost and longer operation time.^[4]^

Although vertebral augmentation therapy is a minimally invasive procedure, it is not free of complications and side effects.^[13–16]^ Among all the iatrogenic reasons in our study, cement leakage to spinal canal accounts for the most common complain. Previous studies demonstrated that the incidence of cement leakage vary from less than 5% to more than 80% of all performed vertebral augmentation procedures.^[17–20]^ Although most leakage was clinically asymptomatic, serious complications occurred in 3.9% to 7.5% of the patients.^[14]^ Combined with the high percentage of cases complained with incomplete informed consent in our study, we suggested that informed consent should involve discussion of all potential risks, especially the risk of cement leakage, during the procedure. Sound and clear communication and complete documentation can help reducing the risk of litigation and protect the doctors if perceived negligence occurs.

On the other hand, nearly 20% of cases complained with unsatisfactory outcome for back pain control. It was believed that bone cement injected into the fractured vertebra during this type of procedure fused the fragments of the vertebra together, and thereby reducing bone pain. However, sometimes patient’s back pain have multiple origins, such as spinous process, fractured vertebra or soft tissue.^[21,22]^ Hence, we suggest that PVP/PKP intervention should be carried out until local back pain persists after 3 weeks of conservative treatment according to the international literatures.^[23–25]^ What’s more, a discussion of the possible residual pain should be noticed in the informed consent to avoid an irrational expectation.

Surprisingly, only 8.3% claims were closed in favor of the defendant doctors in current study, which is extreme low. It seems that doctors in China are more likely to lose the lawsuit compared with their western country counterparts. This may be explained by the differences in providing evidences among medical malpractice litigation in Chinese legal systems. For a patient/plaintiff to win a malpractice litigation suit in western countries, he/she must demonstrate that a physician was negligent. However things are far different in China since 2002 when the People’s Supreme Court issued a regulation to invert the evidential burden in medical malpractice litigation, aimed improving the situation of information asymmetry between patient and physician. In other words, the defendant doctor needs to demonstrate him/herself not guilty by providing full and complete documentation in malpractice litigation in China.

Malpractice litigation is costly from a financial perspective, and its monetary burden on healthcare systems worldwide keeps rising.^[26–30]^ For the entire included cases in current study, the average verdict payout (331,448 Yuan, around 47,350 US dollars) is significant lower than the average plaintiff claimed payout (861,884 Yuan, around 123,126 US dollars) (*P* < .05). This number is significant lower than the average compensation involving spine surgery addressed by reports from other countries.^[5–7,26]^ On the other hand, a significant longer time from surgery to verdict was observed in claims closed in favor of the defendant doctors, compared with that closed in favor of the plaintiff (3.6 years vs 2.2 years). Showing that doctors in China are at a disadvantage and it would be a long-running twist of bitter for them to win a lawsuit involving vertebral augmentation therapy.

We are fully aware of possible limitations. First of all, although court records are available via legal databases, reporting of cases is not mandatory and is at the discretion of individual court systems and judges. The cases captured may not represent all of the cases in China and may not represent the true distribution of the different reasons for lawsuits and decisions. Secondly, the databases contain only cases with a court verdict and do not include cases that were dropped/discarded at an earlier stage. Out-of-court settlements may not have been filed under court records. Given that a majority of claims do not end into trial, it is unsurprising that our analysis may only captured a fraction of the total number of claims in China. Finally, the information presented in these cases is primarily legal in nature and medical details from most cases are not publicly reported, which limits the available information on claims.

In conclusion, there is a quickly rising trend in the number of medical malpractice litigation involving vertebral augmentation therapy in China. Claims were closed in favor of the plaintiff in the majority of cases. Identifying the most common reasons for litigation and summarizing their characteristics may help decrease litigation rate and improve the patient experience.

## Acknowledgments

We appreciate Shiqi Dong from King & Wood Mallesons for her kindness of legal advises.

## Author contributions

**Conceptualization:** Songhua Xiao.

**Data curation:** Dong Hu, Huawei Liu, Bei Yuan, Kai Xu.

**Formal analysis:** Dong Hu.

**Investigation:** Fei Song.

**Methodology:** Dong Hu.

**Supervision:** Fei Song, Songhua Xiao.

**Writing – original draft:** Dong Hu.

**Writing – review & editing:** Huawei Liu, Suxi Gu, Songhua Xiao.
